# Impact of National Lockdown on the Hyperacute Stroke Care and Rapid Transient Ischaemic Attack Outpatient Service in a Comprehensive Tertiary Stroke Centre During the COVID-19 Pandemic

**DOI:** 10.3389/fneur.2021.627493

**Published:** 2021-02-11

**Authors:** Lucio D'Anna, Maddison Brown, Sikdar Oishi, Natalya Ellis, Zoe Brown, Paul Bentley, Brian Drumm, Omid Halse, Sohaa Jamil, Harri Jenkins, Abid Malik, Dheeraj Kalladka, Marius Venter, Joseph Kwan, Soma Banerjee

**Affiliations:** ^1^Department of Stroke and Neuroscience, Charing Cross Hospital, Imperial College London NHS Healthcare Trust, London, United Kingdom; ^2^Department of Brain Sciences, Imperial College London, London, United Kingdom

**Keywords:** COVID-19, stroke, transient ischaemic attack, thrombolisis, stroke care, lock down

## Abstract

**Background:** The COVID-19 pandemic is having major implications for stroke services worldwide. We aimed to study the impact of the national lockdown period during the COVID-19 outbreak on stroke and transient ischemic attack (TIA) care in London, UK.

**Methods:** We retrospectively analyzed data from a quality improvement registry of consecutive patients presenting with acute ischemic stroke and TIA to the Stroke Department, Imperial College Health Care Trust London during the national lockdown period (between March 23rd and 30th June 2020). As controls, we evaluated the clinical reports and stroke quality metrics of patients presenting with stroke or TIA in the same period of 2019.

**Results:** Between March 23rd and 30th June 2020, we documented a fall in the number of stroke admissions by 31.33% and of TIA outpatient referrals by 24.44% compared to the same period in 2019. During the lockdown, we observed a significant increase in symptom onset-to-door time in patients presenting with stroke (median = 240 vs. 160 min, *p* = 0.020) and TIA (median = 3 vs. 0 days, *p* = 0.002) and a significant reduction in the total number of patients thrombolysed [27 (11.49%) vs. 46 (16.25%, *p* = 0.030)]. Patients in the 2020 cohort presented with a lower median pre-stroke mRS (*p* = 0.015), but an increased NIHSS (*p* = 0.002). We registered a marked decrease in mimic diagnoses compared to the same period of 2019. Statistically significant differences were found between the COVID and pre-COVID cohorts in the time from onset to door (median 99 vs. 88 min, *p* = 0.026) and from onset to needle (median 148 vs. 126 min, *p* = 0.036) for thrombolysis whilst we did not observe any significant delay to reperfusion therapies (door-to-needle and door-to-groin puncture time).

**Conclusions:** National lockdown in the UK due to the COVID-19 pandemic was associated with a significant decrease in acute stroke admission and TIA evaluations at our stroke center. Moreover, a lower proportion of acute stroke patients in the pandemic cohort benefited from reperfusion therapy. Further research is needed to evaluate the long-term effects of the pandemic on stroke care.

## Introduction

The Coronavirus disease 2019 (COVID-19) outbreak began in December 2019 in Wuhan, Hubei province, China and then spread to Europe in January 2020 (1). The index case entered the United Kingdom (UK) on January 23rd 2020 from Hubei province in China (2). Subsequently, unique measures such as large-scale application of social isolation, closing borders and nationwide lockdown were adopted in UK since March 23, throughout June 2020, to fight against COVID-19.

The COVID-19 outbreak has led to a huge reorganization of the health care systems worldwide and unprecedent strategies were rapidly implemented to face the increasing needs for COVID-19 patient such as resource allocation, mobilizing workforce, and optimizing bed availability (4). As a result several groups reported that stroke care suffered from a shortage of services and delays in time-dependent treatments and diagnostic work-up since the onset of the pandemic (3, 5–9). In addition, at the same time, observational studies showed a marked and unexplained reduction in the number of patients admitted in hospital with cardiovascular pathologies such as myocardial infarction and acute ischaemic stroke (3, 10–32). However, these preliminary global reports explored mainly the impact of the pandemic only on the overall volume of hospital admissions for acute stroke but with no report about the implications on the outpatient rapid Transient Ischaemic Attack (TIA) services.

In this study we sought to investigate the impact of the national lockdown measures during the COVID-19 pandemic on the rate of admission of stroke patients to the Hyper Acute Stroke Unit (HASU) and rate of patients evaluated in the rapid outpatient TIA service of a comprehensive tertiary stroke center in London (UK) compared to a pre-pandemic cohort. We also investigated clinical characteristics of the patients, stroke reperfusion therapies and treatment metrics.

## Methods

This was a an observational, retrospective, single-center study based on data of consecutive patients with acute stroke and transient ischaemic attack (TIA) admitted to the Hyper Acute Stroke Unit (HASU) or evaluated in the rapid outpatient TIA service of the Stroke Department, Charing Cross Hospital, Imperial College Health Care Trust London between March 23rd and 30th June 2019 and between March 23rd and 30th June 2020. The Stroke Department at Charing Cross Hospital is a comprehensive tertiary stroke center and is the North West London (UK) regional lead referral stroke center for mechanical thrombectomy for a population of over 6.4 million people. It cares for over 1,800 patients admitted to the HASU annually and over 900 patients assessed in the rapid outpatient TIA service annually. The 24/7 thrombectomy service treats stroke patients presenting within 6 h of symptom onset, as well as selected patients with wake-up stroke (unclear time of onset) or presenting between 6 to 24 h using computed tomography (CT) perfusion imaging protocols.

**Figure 1:**
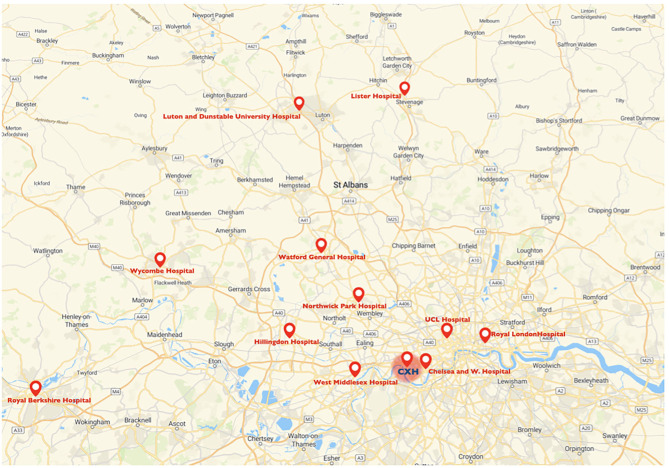
Map of the catchment area for mechanical thrombectomy of the Charing Cross Hospital and referring centers. CXH, Charing Cross Hospital.

The Charing Cross Hospital as lead referral stroke center for mechanical thrombectomy accepts potential candidate for mechanical thrombectomy from the hyper acute stroke units of Luton and Dunstable University Hospital, Lister Hospital, Watford General Hospital, Northwick Park Hospital, Royal Berkshire Hospital, Wycombe Hospital, Royal London Hospital and University College London Hospital; but also from the acute stroke units at Chelsea and Westminster Hospital, Hillingdon Hospital and West Middlesex Hospital (Figure 1). Patients are accepted following a telephone consultation between the referring center and the on-call stroke consultant. The Charing Cross Hospital began performing diagnostic nasopharyngeal swabs for SARS-CoV-2 virus from March 3, 2020. Only between the 18th March and 30th April 2020, external stroke patients, proven to have COVID-19, were not transferred for thrombectomy at our hospital, but otherwise all external referral hospitals were instructed to continue referring patients for thrombectomy, including those with suspected COVID-19, via the same process as before the COVID-19 pandemic. The Thrombectomy management board released a new modified COVID stroke thrombectomy pathway with the aim to protect frontline health-care staff, reduce footprint across the hospital and maintain communication between team members (33). The primary outcome measure was to study the overall volume of patients admitted to our HASU and patients evaluated in our rapid outpatient TIA service between March 23rd and 30th June 2020 compared to the same period in 2019. The secondary clinical outcomes were to investigate patient demographics, clinical characteristics, proportion of acute recanalization therapy performed, stroke treatment metrics and final diagnosis between the two groups of patients (2020 vs. 2019).

### Data Source and Data Collection Process

A database of admissions that is used for reporting to a central UK stroke data bank (Sentinel Stroke National Audit Programme) ensured the consecutive enrolment of eligible patients. Electronic medical records of eligible patients were obtained from the Imperial College Healthcare NHS Trust medical archive. Data of consecutive patients were extracted using a pre-specified case report file that included patient characteristics, including age, vascular risk factors and relevant medical history that were recorded during the admission. Events were captured by review of medical notes of all patients admitted to the HASU and referred to the rapid outpatient TIA service of the Imperial College Healthcare NHS Trust between March 23rd and 30th June 2020 and between March 23rd and 30th June 2019.

### Definition of Study Variables

Ischaemic stroke was defined as an episode of neurological dysfunction caused by focal cerebral, spinal or retinal infarction (34). TIA was defined as a brief episode of neurological dysfunction caused by focal brain or retinal ischemia, with clinical symptoms typically lasting <1 h, and without evidence of acute infarction (35). Intracerebral hemorrhage (ICH) refers to primary, spontaneous, non-traumatic bleeding occurring in the brain parenchyma (36). Cerebral venous thrombosis refers to thrombosis of the dural sinus and/or cerebral veins (CSVT) (37). Stroke mimics included migraine aura, seizures, syncope, peripheral vestibular disturbance, transient global amnesia, functional/anxiety disorder, amyloid spells, subarachnoid hemorrhage, structural brain lesion and paroxysmal symptoms due to demyelination (38). The severity of the index stroke was assessed using the National Institutes of Health Stroke Scale (NIHSS) score on admission. The modified Rankin Scale (mRS) was used to assess patient's initial premorbid status pre-stroke and level of functional independence at 90 days of the patients who underwent mechanical thrombectomy. Data included point of first healthcare provider contact (999/F.A.S.T., emergency department or ED, local general practitioners or GP, etc). Data on known stroke risk factors were collected as follows: age, sex, current cigarette smoking, history of hypertension (blood pressure >140/90 mm Hg at least twice before acute stroke or already under treatment with antihypertensive drugs), history of diabetes mellitus (a random venous plasma glucose concentration >11.1 mmol/l or a fasting plasma glucose concentration >7.0 mmol/l or 2 h plasma glucose concentration >11.1 mmol/l 2 h after 75 g anhydrous glucose in an oral glucose tolerance test, or HbA1c >48 mmol/mol or under antidiabetic treatment), history of dementia, history of symptomatic ischemic heart disease (myocardial infarction, history of angina, or previous diagnosis of multiple lesions on thallium heart isotope scan or evidence of coronary disease on coronary angiography), history of symptomatic peripheral arterial disease (intermittent claudication of presumed atherosclerotic origin; or ankle/arm systolic blood pressure ratio <0.85 in either leg at rest, or history of intermittent claudication with previous leg amputation, reconstructive surgery, or angioplasty), previous stroke/ TIA and previous ICH. Process time variables were collected prospectively, when applicable, and included door to needle time, door to computer tomography (CT) time, CT to decision time and onset to needle time for intravenous thrombolysis (IVT); and door to groin puncture time and onset to groin puncture time for endovascular thrombectomy (EVT).

### Brief Description of the Workflow

Patients presenting with features of acute stroke were evaluated in the hyperacute setting with appropriate neuroimaging and vascular imaging when indicated: CT, computed tomography angiography (CTA), computed tomography perfusion (CTP) of the brain and magnetic resonance imaging (MRI). Patients who fulfilled the relevant indications and without exclusion criteria would undergo acute recanalization therapy. Eligible patients who presented up to 4.5 h of ischaemic stroke symptoms onset received IVT with recombinant-tissue plasminogen activator (r-TPA) (39). Stroke patients would be considered for endovascular thrombectomy (EVT) if they met the following criteria: pre-stroke mRS 0–2, NIHSS score 6 or more, Alberta Stroke Program Early CT score (ASPECTS) 5 or more and within 6 h of symptom onset, anterior circulation large vessel occlusion, basilar artery occlusion. Selected AIS patients within 6 to 24 h of last known normal may be included if they meet other DAWN or DEFUSE 3 eligibility criteria (39–41).

Local GPs in primary-care or Emergency Departments (ED) can refer any patient they suspect had a TIA, but whom they did not consider required immediate hospital admission, to our rapid outpatient TIA service. These patients or the caregiver at home (usually by telephone) are then contacted by our team to arrange a clinic appointment within 24 h of referral received. Our TIA clinic is organized to provide a standardized assessment to all our patients. On the same day, blood tests, ECG, brain imaging (usually CT), and carotid ultrasound imaging and a clinical assessment by a stroke physician are obtained. Patients are discharged home immediately after the assessment, unless the treating physician believes the patient requires urgent admission to our HASU.

### Study Outcomes and Statistical Analysis

Continuous variables are presented as mean with standard deviation (sd) if values are normally distributed or as median with interquartile range (IQR) when they do not follow the normal distribution. We compared the distribution of continuous variables between groups with *t*-test or Wilcoxon rank-sum test as appropriate, whereas categorical values were compared with chi-square tests. Statistical significance was set at 0.05. All analyses were conducted with Stata 15.1 (StataCorp, College Station, TX).

## Results

### Hyperacute Stroke Care

Between the March 23rd and June 30th, 2019, we admitted 514 patients in our HASU while we documented 353 admissions between the March 23rd and June 30th, 2020. This represents a fall in admissions of 31.33%. In Table 1 we showed the clinical characteristics of the two groups of patients admitted during the two study periods. There were no statistically significant differences with regards to age and gender distribution. However, patients admitted during the COVID-19 pandemic showed lower pre-stroke mRS scores (*p* = 0.015) and higher median NIHSS on arrival (*p* = 0.002) compared to the patients admitted in same period in 2019. Moreover, the median symptom onset-to-door time was significantly longer (*p* = 0.020) and the median length of inpatient stay in HASU was increased (*p* < 0.001) in the group of patients admitted between the 23rd March and 30th June 2020 compared to the same period in 2019.

**Table 1:** Clinical characteristics of the two cohorts of patients admitted in HASU during the two study periods.

	**23rd March to 30th June 2020 (*n* = 353)**	**23rd March to 30th June 2019 (*n* = 514)**	***P***
Age, y (median, IQR)	70.5; 35–98	71.0; 24–96	0.863
Male, sex (%)	196 (56.97%)	250 (49.70%)	0.134
mRS pre-stroke (median, IQR)	0; 0–5	1; 0–4	0.015
NIHSS on arrival (median, IQR)	7; 0–30	4; 0–29	0.002
Symptom onset -to-door time, min (median, IQR)	240; 20–10,080	160; 27–23,040	0.020
Length of inpatient stay in HASU, days (median, IQR)	4; 1–60	2; 0–20	<0.001
**First healthcare provider contacts used by stroke patients**			
GP	69 (20.05%)	30 (5.96%)	
ED	172 (50%)	216 (42.94%)	
999/F.A.S.T. emergency call	103 (29.95%)	257 (51.1%)	
			<0.001
**Final diagnosis**			
Ischaemic stroke	235 (66.57%)	283 (55.06%)	
Intracranial hemorrhage	41(11.61%)	48 (9.34%)	
TIA	18 (5.1%)	49 (9.53%)	
CSVT	4 (1.13%)	2 (0.39%)	
Stroke mimic	55 (15.58%)	132 (25.68%)	
			<0.001

*mRS, modified Rankin Scale; IQR, interquartile range; HASU, hyper acute stroke unit; GP, general practitioner; ED, emergency department; F.A.S.T., face, arm, speech, time; TIA, transient ischemic attack; CSVT, cerebral sinus venous thrombosis*.

We documented a statistically significant difference in terms of the first medical provider contact used by the stroke patients during the two study periods (Table 1) (*p* < 0.001). Between the 23^rd^ March and 30^th^ June 2020, 20.05% (vs. 5.96% in 2019) of the patients had their local GP as first medical provider contact while 29.95% (vs. 51.1% in 2019) of the patients used the emergency medical service 999/F.A.S.T.

**Figure 2:**
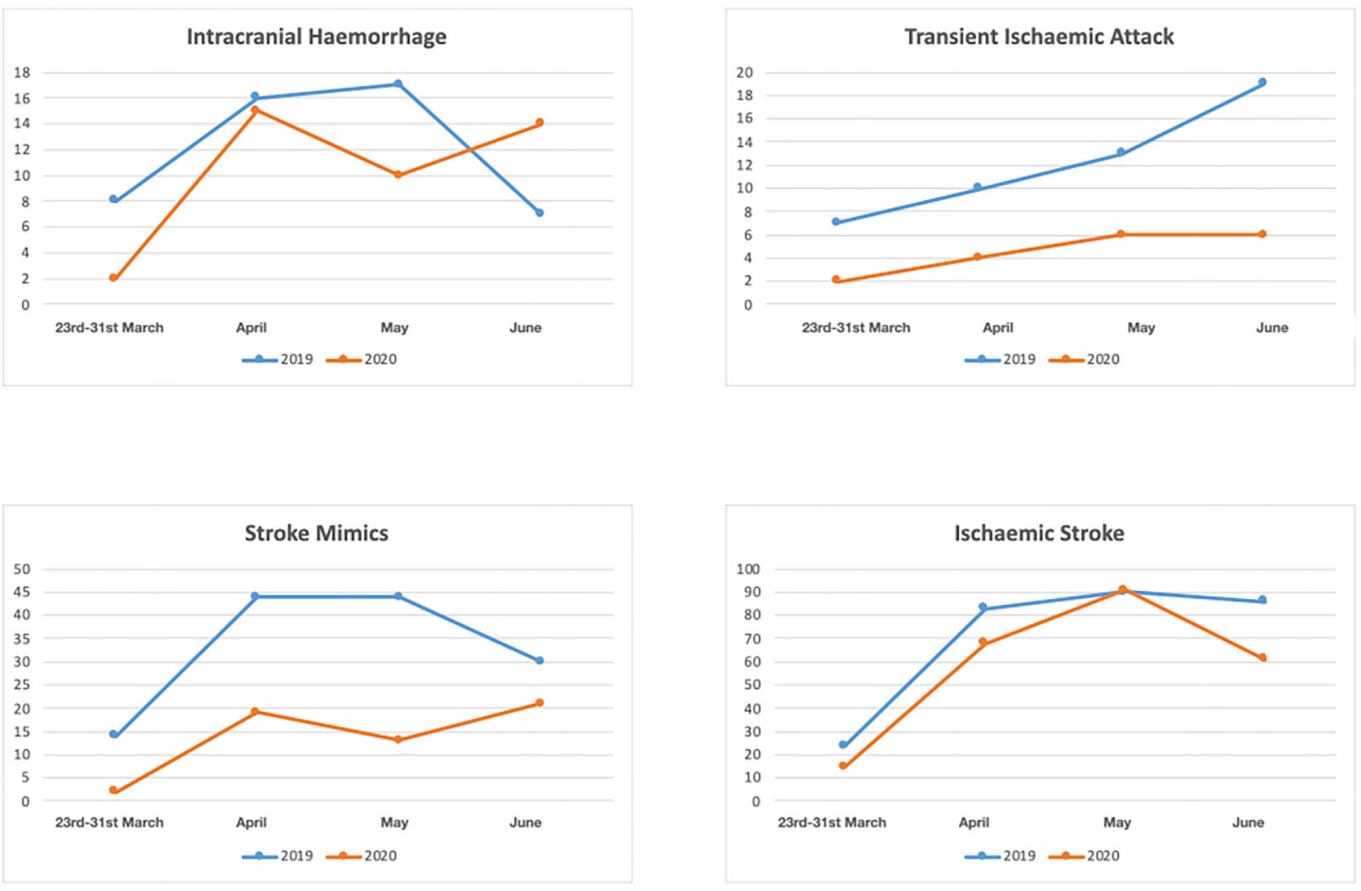
Numbers of patients with diagnosis of intracranial hemorrhage, transient ischaemic attack, stroke mimic and ischaemic stroke between March 23rd and 30th June 2020 and between March 23 and 30 June 2019.

Between the 23rd March and 30th June 2020, the final diagnosis was ischemic stroke in 235 (66.57%), intracranial hemorrhage in 41(11.61%), TIA in 18 (5.1%), CSVT in 4 (1.13%) and stroke mimic in 55 (15.58%); in the same period in 2019 the final diagnosis was ischemic stroke in 283 (55.06%), intracranial hemorrhage in 48(9.34%), TIA in 49 (9.53%), CSVT in 2 (0.39%) and stroke mimic in 132 (25.68%) (*p* < 0.001) (Table 1) (Figure 2).

**Figure 3:**
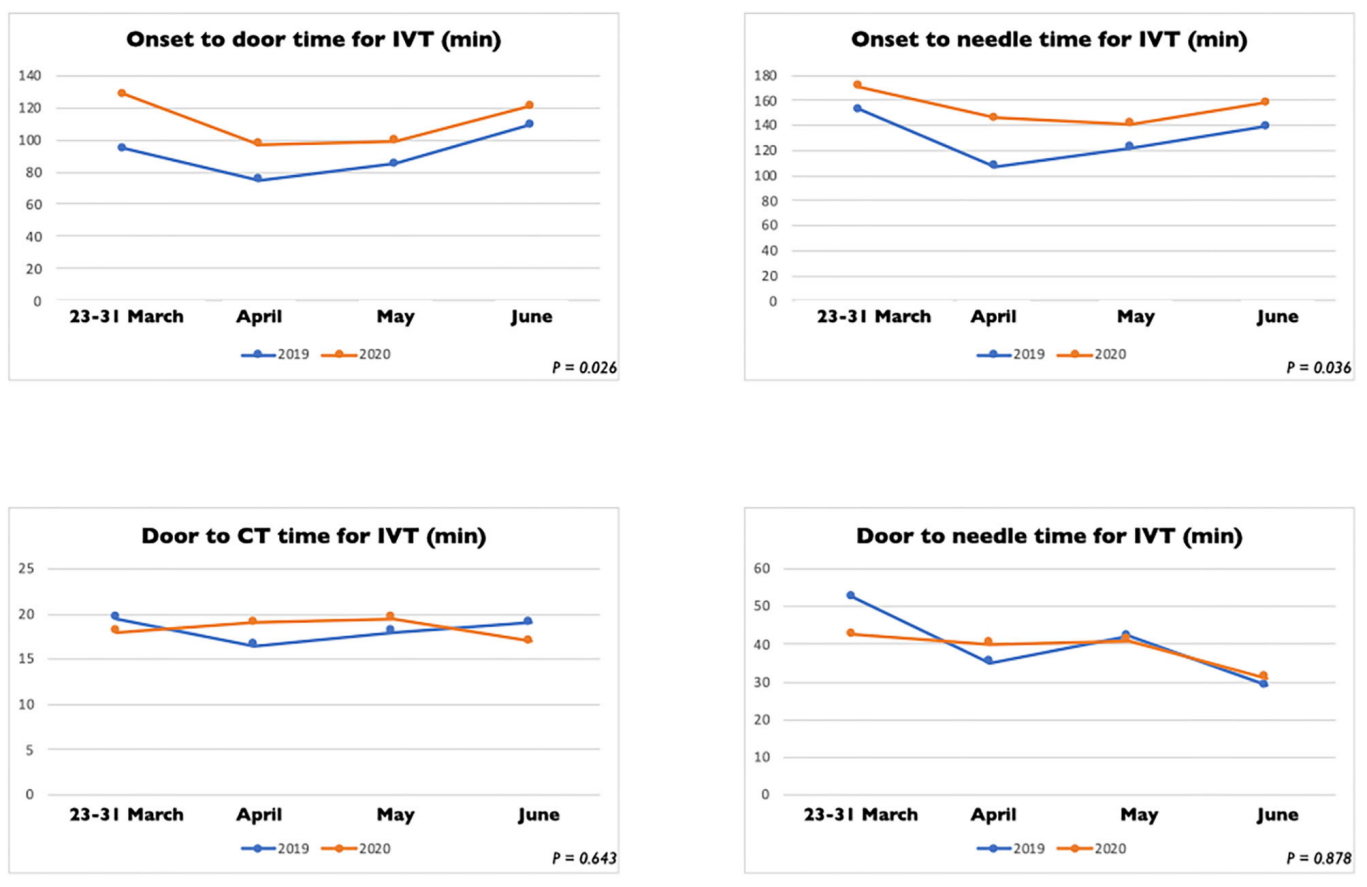
Process measure comparison for intravenous thrombolysis between stroke patients admitted between March 23 and 30 June 2020 and between March 23 and 30 June 2019.

In Table 2 we reported the clinical characteristics and type of acute treatments received by the two groups of patients with ischaemic stroke admitted in our HASU. Patients' clinical characteristics were similar in both groups but patients admitted in the 2020 cohort more frequently had diabetes (*p* = 0.031) and less frequently had a past history of dementia (*p* = 0.042), or intracranial hemorrhage (*p* = 0.017). Regarding the acute treatment received, patients admitted between 23rd March and 30th June 2020 less frequently underwent IVT alone (*p* = 0.030) while more frequently were treated with IVT combined with EVT (*p* = 0.043). There was no significant difference between the two cohorts of patients treated with IVT alone, EVT alone or IVT plus EVT in terms of NIHSS on arrival and 24h NIHSS. In terms of process measures time (Figure 3), statistically significant differences were found between the COVID and pre-COVID cohorts in the time from onset to door arrival (median 99 vs. 88 min, *p* = 0.026) and from onset to needle time (median 148 vs. 126 min, *p* = 0.036) for IVT. We did not observe significant difference in the door to CT time, CT to decision time, door to needle time for IVT and door to groin puncture time and onset to groin puncture time for EVT. The median mRS at 90 days for the patients treated with EVT (alone or combined with IVT) did not differ among the two cohorts of patients (*p* = 0.403); moreover, we did not find any statistically significant difference between the two groups of patients regarding the proportion of patients who received EVT able to achieve functional independence (mRS score of 0-2) at 90 days (*p* = 0.367) (Table 3).

**Table 2:** Clinical characteristics, reperfusion therapy rate and process measures of the patients with ischaemic stroke admitted in HASU during the two study periods.

	**23rd March to 30th June 2020 (*n* = 235)**	**23rd March to 30th June 2019 (*n* = 283)**	***P***
Age, y (median, IQR)	78; 72–83	80; 73–85	0.236
Male, sex (%)	127 (54.04%)	157 (55.48%)	0.468
Smoking, *n* (%)	44 (18.72%)	53 (18.72%)	0.997
Hypertension, *n* (%)	143 (60.85%)	158 (55.83%)	0.420
Diabetes, *n* (%)	66 (28.09%)	47 (16.61%)	0.031
Dementia, *n* (%)	7 (2.98%)	23 (8.12%)	0.042
Coronary artery disease, *n* (%)	19 (8.09%)	20 (7.07%)	0.867
Heart failure, *n* (%)	12 (5.11%)	18 (6.36%)	0.694
Peripheral vascular disease, *n* (%)	8 (3.40%)	6 (2.12%)	0.537
Previous ischemic stroke/TIA, *n* (%)	61 (25.96%)	85 (30.04%)	0.488
Previous ICH, *n* (%)	3 (1.27%)	22 (7.77%)	0.017
Previously known AF, *n* (%)	61 (25.96%)	51 (18.02%)	0.128
**Reperfusion therapy**			
**IVT alone**, ***n*** **(%)**	**27 (11.49%)**	**46 (16.25%)**	**0.030**
NIHSS on arrival for IVT (median, IQR)	7.5; 2–22	8; 2–25	0.546
24 h NIHSS for IVT (median, IQR)	2; 0–22	2; 0–33	0.640
**EVT alone**, ***n*** **(%)**	**13 (5.53%)**	**11 (3.89%)**	**0.879**
NIHSS on arrival for EVT (median, IQR)	17; 11–25	18; 2–29	0.857
24 h NIHSS for EVT (median, IQR)	18; 1–30	14.5; 1–38	0.640
**IVT and EVT**, ***n*** **(%)**	**44 (18.72%)**	**33 (11.66%)**	**0.043**
NIHSS on arrival for IVT and EVT (median, IQR)	18.5; 0–27	17; 7–28	0.924
24 h NIHSS for IVT and EVT (median, IQR)	12.5; 0–38	9; 0–37	0.174
**Process measures**			
Onset to door time for IVT, min (median, IQR)	99; 44–265	88; 15–386	0.026
Onset to needle time for IVT, min (median, IQR)	148; 46–327	126; 36–190	0.036
Door to CT time for IVT, min (median, IQR)	19; 2–50	17; 6–53	0.643
CT to decision time for IVT, min (median, IQR)	18; 1–83	12; 1–72	0.155
Door to needle time for IVT, min (median, IQR)	39; 33.5–45	40; 34–44.5	0.878
Door-to-groin puncture time for EVT, min (median, IQR)	58; 20–325	55; 21–289	0.982
Onset-to-groin puncture time for EVT, min (median, IQR)	143; 68–275	122; 71–473	0.701

*TIA, transient ischemic attack; ICH, intracranial hemorrhage; AF, atrial fibrillation; IQR, interquartile range; IVT, intravenous thrombolysis; EVT, endovascular treatment; CT, computer tomography*.

**Table 3:** Efficacy outcomes of the patients who underwent EVT (alone or combined with IVT) during the two study periods.

**Outcome**	**23^**rd**^ March to 30^**th**^ June 2020 (*n* = 57)**	**23^**rd**^ March to 30^**th**^ June 2019 (*n* = 44)**	***P***
mRS at 90 days, (median, IQR)	3; 0–6	3; 0–6	0.403
Functional independence at 90 days [mRS 0–2, *n* (%)]	20 (35.09%)	15 (34.09%)	0.367
Death at 90 days	12 (21.05%)	15 (34.09%)	0.283

*mRS, modified Rankin Scale; IQR, interquartile range*.

### TIA Rapid Outpatient Service

Between the 23rd March and 30th June 2019, 180 patients were referred with suspected TIA to our rapid TIA outpatient service while 136 patients were referred in same period in 2020. This represents a fall in the number of referrals by 24.44%. Patients characteristics were similar in both groups, but patients referred during the COVID-19 period had less frequently dementia (*p* = 0.027) (Table 4). The median symptom onset-to-first medical review time was significantly longer in the group of patients referred between the 23rd March and 30th June 2020 compared to the same period in 2019 (median = 3 vs. 0 days, *p* = 0.002).

**Table 4:** Baseline characteristics of the patients evaluated in the rapid outpatient TIA service during the two study periods.

	**23^**rd**^ March to 30^**th**^ June 2020 (*n* = 136)**	**23^**rd**^ March to 30^**th**^ June 2019 (*n* = 180)**	***P***
**Demographics**			
Age, y (median, IQR)	65; 23–96	68.5; 20–28	0.087
Male, sex, *n* (%)	99 (72.79%)	121 (67.22%)	0.227
**Clinical characteristics**			
Hypertension, *n* (%)	71 (52.21%)	95 (52.78%)	0.442
Diabetes mellitus, *n* (%)	27 (19.85%)	37 (20.56%)	0.645
Hypercholesterolemia, *n* (%)	65 (47.79%)	76 (42.22%)	0.638
Coronary artery disease, *n* (%)	19 (13.97%)	21 (11.67%)	0.695
Previous TIA/stroke, *n* (%)	23 (16.91%)	39 (21.67%)	0.186
Carotid stenosis, *n* (%)	3 (2.21%)	9 (5.0%)	0.164
AF, *n* (%)	15 (11.03%)	25 (13.89%)	0.334
Dementia, *n* (%)	6 (4.41%)	18 (10%)	0.027
ABCD2 score, (median, IQR)	3; 0–6	3; 0–6	0.929
Symptom onset-to-first medical review time (days), (median, IQR)	3; 0–90	0; 0–133	0.002
**First healthcare provider contacts used by TIA patients**			
GP, *n* (%)	41 (30.14%)	75 (41.67%)	
ED, *n* (%)	93 (68.38%)	97 (53.89%)	
Other^*^, *n* (%)	2 (1.48%)	6 (3.33%)	
			0.020
**Final diagnosis**			
TIA, *n* (%)	75 (55.15%)	83 (46.11%)	
Ischemic stroke, *n* (%)	13 (9.56%)	9 (5.0%)	
TIA mimic, *n* (%)	48 (35.29%)	88 (48.89%)	
			0.020

*IQR, interquartile range; TIA, transient ischemic attack; AF, atrial fibrillation; GP, general practitioner; ED: emergency department*;

* *this includes referrals from other specialties consultant*.

We documented a statistically significant difference in terms of the first medical provider contact used by the TIA patients during the two study periods (Table 4) (*p* = 0.020). Between the 23^rd^ March and 30^th^ June 2020, 30.14% (vs. 41.67% in 2019) of the patients had their local GP as first medical provider contact while 68.38% (vs. 53.89% in 2019) of the patients self-referred to the ED of our hospital.

Finally, we observed a statistically significant difference in the final diagnosis (*p* = 0.020) (Table 4) after the review in our TIA service. The percentage of patients with a final diagnosis of TIA increased from 46.11% in 2019 to 58.22% during the COVID period.

Interestingly, our ambulatory rapid access TIA clinic experienced a doubling rate of ischaemic stroke diagnoses (9.56 vs. 5.0%), whilst registering a marked decrease in mimic diagnoses (35.29 vs. 48.89%) during the COVID period. Of note, 32.88% of the 2020 cohort had a final diagnosis of TIA mimic whilst in the same period in 2019 this was 48.89%.

## Discussion

In this observational study we explored the impact of the national lockdown measures due to the COVID-19 pandemic on our large regional tertiary stroke center. One element of novelty of our analysis is that investigated the impact of the COVID-19 outbreak on the hyper acute stroke care but also on the rapid outpatient TIA service care at a comprehensive tertiary stroke center. The main finding of our analysis is that our stroke sample presented with a lower median pre-stroke mRS combined with an increase in initial stroke severity during the national lockdown. In addition, our study showed that the proportion of patients with previous history of dementia, admitted to our HASU or assessed in our rapid outpatient TIA clinic, was statistically significantly lower during the COVID-19 outbreak compared to the same period in 2019. Several centers have described significant difference in severity at presentation of stroke patients but to date no difference in the degree of pre-stroke disability or dependence in the daily activities has been reported (11, 30, 32). Based on the available data, poor pre-admission functional status and dementia are risk factors for in-hospital mortality in patients with COVID-19 (42). Our hypothesis is that vulnerable patients and their caregivers might have intentionally avoided hospital admissions due to the risk of COVID-19 infection. Alternatively, epidemic response measures might also represent a contributing factor. Self-isolation reduces social connections, especially in the elderly and more frail population. Isolation can impact on early recognition of stroke symptoms and can lead to delayed notification of emergency services. Indeed, similar to previous studies (31), we have observed a significant delay in symptom onset-to-door review time in our sample that could support this thesis.

Another key finding is that we showed an overall significant reduction in the hospitalization rate for stroke and in the number of patients presenting with TIA. The rate of thrombolysis delivery also reduced. Our results are in line with previous observational studies and have confirmed our preliminary report (43). A survey of 81 Italian stroke centers conducted by the Italian Stroke Organization reported a reduction of about 26–30% in the hospitalization rate for minor stroke and TIA, and of about 50% for stroke acute therapies in comparison with the same period in 2019 (3, 44). In Germany the marked decrease of patients with TIA or minor stroke presenting in hospital has led the German Society of Neurology and the German Stroke Society to initiate a publicity campaign in television and newspapers about the so-called ‘phenomenon of empty stroke units’ to invite patients to seek medical help (3). Similarly, in the USA (30) and in China (5) there are reports of reduction in acute stroke volume in hospitals. This concern has also been raised by the World Stroke Organization (45). There are several factors that could potentially explain this phenomenon. First, fear of in-hospital infection and advice from health authorities, media and doctors probably led patients with mild symptoms to stay at home. Interestingly, our ambulatory rapid access TIA clinic experienced a doubling rate of ischaemic stroke diagnoses, whilst registering a marked decrease in mimic diagnoses. This is in keeping with the hypothesis that milder stroke patients were avoiding hospital admissions due to fear of the pandemic, and preferred an outpatient setting where accessible. This does however delay their presentation and limit access to reperfusion therapies. For this reason, information campaigns to educate patients to present early to the ED if they have symptoms suggestive of stroke must be implemented even during this ongoing COVID-19 pandemic. Secondly, primary-care, EDs and ambulance services have undergone significant pressures due to the volume of COVID-19 patients. This might have induced additional delays and errors during patient triage and transport, thus reducing the proportion of patients eligible for acute treatment. Moreover, the COVID-19 pandemic is having implications on stroke services in all parts of the world in terms of redeployment of stroke staff, and reallocation of the stroke beds to COVID-19 patients (45). Resources management is critical during the pandemic and should be established as quickly as possible. Designated stroke centers should be assigned to maintain resources for delivery of high-quality stroke care (5).

In our center, we observed a significant reduction in the proportion of patients that used the emergency medical service 999/F.A.S.T. after the onset of stroke symptoms compared to the same period in 2019. This is in line with the NHS England data showing that during the COVID-19 pandemic was a general reduction at national level in emergency admissions (46).

This could probably explain the longer delay in symptom onset-to-door time and onset to needle time in stroke patients and consequently the reduced proportion of patients who presented within the therapeutic time window for thrombolysis during the COVID pandemic. Interestingly, we did not observe any significant delay to reperfusion (door-to-needle and groin-to-puncture time) for IVT and EVT, in line with other centers worldwide (11, 13, 22, 27, 30–32) and with our preliminary report (47). By contrast Meza et al. (6) and Briard et al. (7) reported an increment in their door-to-needle time likely secondary to their new in-hospital infection control measures to manage stroke patients with suspected COVID-19 that may have delayed the acute stroke management. Moreover, our analysis showed that after any reperfusion therapy (IVT, EVT and IVT plus EVT) there was no statistically significant difference in terms of early neurology outcome although patients treated during the national lockdown demonstrated to have a higher 24 h NIHSS after the treatment. Despite the unprecedented demands on emergency healthcare, early multidisciplinary efforts to adapt our acute stroke treatment process resulted in keeping the stroke quality time metrics close to the pre-pandemic levels in our center. Future research with larger sample is needed to evaluate the impact of the delayed presentation of stroke patients during the pandemic on long-term outcomes.

Our study has several limitations and strengths. It is limited by its single-center design. Our findings reflect the trend in a determined area which may not be generalized to all international healthcare practices. Although the demographics and clinical characteristics were similar between cohorts, the possibility of systemic or random bias cannot be excluded. The retrospective design is another limitation. Finally, a long-term follow up was not available for analysis. The strengths of our study include that this is the first single-center report to assess the impact of the national lockdown due to the COVID-19 pandemic on both the HASU admissions and the rapid outpatient TIA service of a comprehensive tertiary stroke center in London (UK). The strengths of our study include also our sample size and the length of the study periods.

In conclusion, the national lockdown in the UK due to the COVID-19 pandemic was associated with a significant decrease in acute stroke admission and TIA evaluations at our comprehensive stroke center. In addition, a lower proportion of acute stroke patients in the pandemic cohort benefited from reperfusion therapy, specifically intravenous thrombolysis. More minor ischaemic stroke patients presented to our rapid access TIA clinic. These findings support concerns that the current ongoing pandemic may have negative impact on the acute management of non-COVID-19-related conditions such as acute stroke. Further research is needed to evaluate the long-term effects of the pandemic on population-based acute stroke incidence, hospital stroke and TIA outpatient evaluations volume, treatment metrics, and long-term outcomes.

## Data Availability Statement

The raw data supporting the conclusions of this article will be made available by the authors, without undue reservation.

## Ethics Statement

This study was performed in accordance with the ethical standards as laid down in the 1964 Declaration of Helsinki and its later amendments. Informed consent of subjects was not needed as the data collected for the study were information collected as part of the routine care and only de-identified data were used in the research.

## Author Contributions

LD'A and SB: study concept, statistical analysis, drafting, and critical revision of manuscript. MB, SO, NE, ZB, and BD: data collection, critical revision of manuscript. OH, SJ, HJ, AM, DK, JK, and MV: critical revision of manuscript. All authors contributed to the article and approved the submitted version.

## Conflict of Interest

The authors declare that the research was conducted in the absence of any commercial or financial relationships that could be construed as a potential conflict of interest.
